# On the importance of full-dimensionality in low-energy molecular scattering calculations

**DOI:** 10.1038/srep28449

**Published:** 2016-06-23

**Authors:** Alexandre Faure, Piotr Jankowski, Thierry Stoecklin, Krzysztof Szalewicz

**Affiliations:** 1 Université de Grenoble Alpes, IPAG, F-38000 Grenoble, France; 2 CNRS, IPAG, F-38000 Grenoble, France; 3 Faculty of Chemistry, Nicolaus Copernicus University in Torun, Gagarina 7, PL-87-100 Torun, Poland; 4 Institut des Sciences Moleculaires, Université de Bordeaux, F-33405 Talence, France; 5 Department of Physics and Astronomy, University of Delaware, Newark, DE 19716, USA

## Abstract

Scattering of H_2_ on CO is of great importance in astrophysics and also is a benchmark system for comparing theory to experiment. We present here a new 6-dimensional potential energy surface for the ground electronic state of H_2_-CO with an estimated uncertainty of about 0.6 cm^−1^ in the global minimum region, several times smaller than achieved earlier. This potential has been used in nearly exact 6-dimensional quantum scattering calculations to compute state-to-state cross-sections measured in low-energy crossed-beam experiments. Excellent agreement between theory and experiment has been achieved in all cases. We also show that the fully 6-dimensional approach is not needed with the current accuracy of experimental data since an equally good agreement with experiment was obtained using only a 4-dimensional treatment, which validates the rigid-rotor approach widely used in scattering calculations. This finding, which disagrees with some literature statements, is important since for larger systems full-dimensional scattering calculations are currently not possible.

The H_2_-CO dimer is an important system in several fields of physics, chemistry, and astrophysics. H_2_ is the most abundant and CO is the second most abundant molecule in interstellar space. While it is difficult to directly observe H_2_ in space, especially in cold molecular clouds (because the large rotational constant of H_2_ implies a very weak emission spectrum whereas the high dust optical depth precludes its observation through ultraviolet absorption), observations of CO provide a large fraction of our knowledge about galaxies. In particular, the two lowest rotational transitions of CO are among the best tracers of star formation in dense molecular clouds^1^. The observations of CO also allow the estimation of H_2_ densities and masses in molecular gas. Low density interstellar media, due to low frequency of collisions, cannot be assumed to maintain local thermodynamic equilibrium. The interpretation of all such phenomena requires the knowledge of H_2_-CO inelastic cross-sections, mainly for rotational, but also for vibrational excitations. These cross-sections are difficult to obtain experimentally, but can be accurately computed from first principles.

The first ingredient in an accurate treatment of H_2_-CO scattering is an accurate potential energy surface. In a balanced approach, one has to control the uncertainties from various sources to obtain optimal accuracy of the potential energy surface. The sources of uncertainties are the level of *ab initio* theory used to approximately solve Schrödinger’s equation, the size of basis set employed in such calculations, the dimensionality of the potential energy surface, the number of grid points used (i.e., the number of *ab initio* calculations performed), and the accuracy of the analytic fit to the computed energies. The first calculations of H_2_-CO scattering were performed in 1976 by Green and Thaddeus^2^ in 2-dimensions, i.e., assuming rigid monomers and neglecting the H_2_ angular degrees of freedom. They used an empirical potential energy surface, obtained by scaling the CO-He surface (the latter surface included some *ab initio* input). The first fully *ab initio* potential energy surface in 4-dimensions was published by two of the present authors^3^ in 1998, later followed^4^ by an improved version, also 4-dimensional, in 2005.

The first full-dimensional calculations on the interaction between H_2_ and CO were published by Kobayashi *et al*.^5^ in 2000. These calculations were performed using the coupled-cluster method with Brueckner’s orbitals and up to perturbative triple excitation contributions, CCSD(T), in an augmented triple-zeta quality basis set. The authors did not provide any analytical form of the surface, the *ab initio* interaction energies were only presented on a grid of points in the 5-dimensional coordinate space (the authors found weak dependence on one of the angles and set this angle to zero for all published grid points). Later, Flower^6^ obtained a fit to these data, but only in 4 dimensions, neglecting additionally the dependence on the H-H separation. The dependence of interaction energies on intramolecular coordinates was investigated in ref. 4. More complete full-dimensional calculations were performed in 2012 by two of the present authors and collaborators^7^,^8^, using coupled-cluster methods with up to perturbative quadruple excitations, CCSDT(Q), and very large basis sets with extrapolations to the complete basis set (CBS) limits. For each given set of intermolecular coordinates, the interaction energies were then averaged over the vibrational motion in the monomers. This approach to reduced-dimensionality potential energy surfaces was introduced in ref. 9 and shown to lead to almost negligible errors relative to full-dimensional calculations of van der Waals dimers spectra. In this way, two four-dimensional surfaces were obtained^7^,^8^: for the ground and first excited vibrational states of CO, with H_2_ at the ground vibrational state in both cases (the former surface will be denoted as *V*_12_). These surfaces have been used in several bound-state and scattering calculations^7^,^8^,^10^.

Very recently, the first full-dimensional analytic potential energy surface for H_2_-CO, further denoted by *V*_Y_, has been published by Yang *et al*.^11^. The *ab initio* interaction energies were calculated at the CCSD(T)-F12 level of theory, where F12 denotes the use of explicitly correlated factors in the basis set, with quadruple-zeta quality basis sets. A very large number of grid points, amounting to almost 400,000, were used. The surface was applied in several full-dimensional scattering calculations. These authors performed also four-dimensional scattering calculations using the surface *V*_12_ from refs 7,8. For high-collision-energy inelastic scattering leading to rotational excitations of CO, the two approaches gave essentially equivalent results in comparison to the experiment of ref. 12. For low-energy scattering leading to CO excitations from the ground to the first excited rotational state, comparisons were made to the experiment of ref. 13 and the six-dimensional calculations agreed with experiment better than four-dimensional ones. Yang *et al*. apparently were not aware that the experimental results of ref. 13 were erroneous, see ref. 10. The latter paper compares theoretical cross-sections, computed using the same *V*_12_ potential, to the corrected experiment and an almost perfect agreement has been achieved. Thus, the calculations on the *V*_Y_ surface exhibit in fact fairly large discrepancies relative to the correct experiment, as pointed out recently in ref. 14. The *V*_Y_ surface was also used to compute rates for the deexcitation of CO from the first excited to the ground vibrational state. In this case, significant improvement of agreement with experiment was achieved compared to older calculations.

We have developed a full-dimensional potential energy surface at a higher level of theory than in ref. 11 and applied it to investigate several low-energy scattering processes using full-dimensional dynamics. We carefully test the conclusion of ref. 11 that the full-dimensional treatment is essential for achieving agreement with low-energy experiment. This issue is of significant importance since full-dimensional scattering calculations are not possible for systems larger than diatom–diatom.

## Potential Energy Surface

The six-dimensional grid in the ***XY*** = (*R*, *θ*_1_, *θ*_2_, *ϕ*, *r*, *s*) coordinates was obtained as a product of the ***X*** = (*R*, *θ*_1_, *θ*_2_, *ϕ*) and ***Y*** = (*r*, *s*) grids, where *R* denotes the distance between the centers of mass (COM) of monomers (the COM of H_2_ is placed at the origin of the coordinate system and the COM of CO at *z* = *R*), *θ*_1_ (*θ*_2_) denote the angle between the 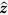 axis and the vector starting at the appropriate COM and ending at H (C), *ϕ* is the dihedral angle between these vectors, whereas *r* and *s* are the interatomic separations in H_2_ and CO, respectively. The details of the grid are given in the Supplementary Information (SI). We have computed interaction energies at 37,894 grid points, but 1,922 of these points were not used since the interaction energies were above 1000 cm^−1^ (such high-energy points would bias the fit if used). To check the quality of our surface above this threshold, we computed the root-mean-square error (RMSE) for the *ab initio* energies which were not used in the fit. For the 1042 such energies in the range 1000 to 2000 cm^−1^, the RMSE is equal to 22.6 cm^−1^, which amounts to 1–2% of the total interaction energy. For the 542 energies larger than 2000 cm^−1^ and smaller than 3000 cm^−1^, the RMSE is equal to 74.7 cm^−1^, which gives a still reasonable 2–4% uncertainty of the fit. Furthermore, 8,840 points were used only to fit the asymptotic part of the energy (the total number of points used in the asymptotic fit was 16,822, i.e., 7,982 points were used both for asymptotics and short-range). Thus, the final potential was fitted to 27,132 points. We have computed an additional 200 *ab initio* interaction energies at randomly generated grid points for testing purposes. The accuracy of the fit will be discussed below and in the SI.

For all the 37,894 points, the interaction energy was computed as


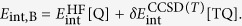


where “B” in the subscript designates the ‘base’ level of theory and basis set, HF stands for the Hartree-Fock method, the letters T and Q denote the use of the aug-cc-pV*X*Z basis sets^15^ with *X* = 3 and 4, respectively, and TQ indicates a CBS extrapolation. At the grid points with intramonomer coordinates (*r*, *s*) = (*r*_*c*_, *s*_*c*_) = (1.474, 2.165) bohr, we have performed calculations at a higher level of theory: CCSD(T) calculations in the aug-cc-pV5Z basis set and CCSDT(Q) calculations in the aug-cc-pVDZ basis set, giving two corrections to *E*_int,B_:


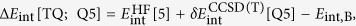


and


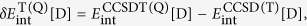


so that the interaction energy at the high-level of theory is defined as


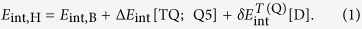


See SI for more details on electronic structure calculations.

To combine the two sets of results, we have employed the idea of the hybrid potential introduced in refs 16, 17, 18 (also indirectly in refs 7,8). In contrast to the previous work, we applied this idea to individual grid points


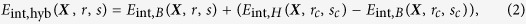


rather than to fitted potential surfaces. Note that for nonlinear monomers, the definition depends on the embedding of monomers in the dimer^18^. Equation (2) was used to fit a 6-dimensional potential. The hybrid Ansatz of Eq. (2) was shown to work well for the water dimer, as the spectra of this system computed in ref. 16 agreed overall better with experiments than the spectra calculated with the base potentials in ref. 19. In the SI, we present data showing that this Ansatz improves accuracy for about 80% of points, most importantly for all points lying near (*r*_*c*_, *s*_*c*_).

## Analytic Fit

The analytic representation of the 6-dimensional (6D) potential energy surface is a generalization of the 4-dimensional (4D) surfaces of the type developed in refs 3,4,8,20 in the sense that the parameters of the intermolecular part are expanded in polynomials of *r* and *s*. The surfaces of this type consist of short-range and long-range (asymptotic) components *V*_sh_ and *V*_as_, respectively. The former component is a sum of powers of *R* multiplied by exponentials of *R*. The latter component is a sum of inverse powers of *R* multiplied by damping functions. In both cases, the linear and nonlinear parameters are dependent on all other coordinates. The coefficients of the asymptotic part for the 4-dimensional case were computed *ab initio* in ref. 3 from monomers’ multipole moments and polarizabilities (static and dynamic). The levels of the asymptotic expansion and of the symmetry-adapted perturbation theory (SAPT)^21^, the same as used in ref. 20, were chosen to enable seamless connection at large values of *R*. The dimensionality, level of theory, and size of the basis set used in ref. 3 are different from those of the present work. Since the asymptotics for the CCSDT(Q) level of theory is unknown, we adopted the expansion of ref. 3, but multiplied each set of terms with a given power of 1/*R* by a factor dependent on all coordinates but *R*. The linear coefficients in these factors were optimized on a set of all interaction energies with *R* ≥ 10 bohr. Such *V*_as_ was then used with frozen coefficients (except for parameters in the damping function) in the optimization of the global potential. The overall RMSE of the fit for all 27,132 grid points is 0.63 cm^−1^, whereas the RMSE on points with negative energies is 0.16 cm^−1^. See SI for details of the fitting procedure.

The procedure described above leads to the total *interaction* potential energy surface *V*(***XY***) = *V*_sh_(***XY***) + *V*_as_(***XY***), further denoted as *V*_15_. To obtain the *total* potential energy surface, one has to add monomer potentials:


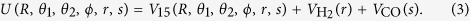


The 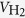 potential for H_2_ was taken from ref. 22, whereas the *V*_C*O*_ potential for CO was obtained by the Rydberg-Klein-Rees procedure in ref. 23 using the measurements of Le Floch^24^.

## Comparison of Surfaces

Our methodology differs in many respects from that used to develop the *V*_Y_ surface in ref. 11, as summarized in Table 1. As one can see, the ranges of coordinates explored are about the same in both cases, however, Yang *et al*.^11^ used a much denser grid, resulting in about 15 times more grid points than in our work. In our opinion, such a dense grid is not needed. As discussed in the SI, tests on randomly chosen grid points not used in the fitting process show that our potential recovers such points sufficiently well, i.e., although the RMSE is larger than on the training set, it is still smaller than the estimated uncertainty of *ab initio* points. Next, we have used a lower energy cutoff than in ref. 11. This actually does not make much difference since in the range of coordinates chosen by us there is only a small number of points with energies above 1000 cm^−1^. In the case of ref. 11, the range of *R* between 4 and 5 bohr, which was only partly covered by us, contains many high-energy points. We believe, however, that this highly repulsive region is not relevant for any of the physical phenomena investigated here. As discussed earlier, our potential reproduces energies in the range 1000–3000 cm^−1^ with errors smaller than 4%.

The functional form of ref. 11 was quite different from ours. Apparently, both forms are capable of fitting the set of computed energies well. Although the RMSE of our fit, 0.63 cm^−1^, is much smaller than that of *V*_Y_, 14.22 cm^−1^, the larger latter value is probably mainly due to the large number of points with energies above 1000 cm^−1^ included in ref. 11. The advantage of our fit is that it decays physically at large *R*, whereas the fit of ref. 11 decays exponentially.

We have used a significantly higher level of theory, CCSDT(Q), than that of the CCSD(T) method used in ref. 11. Also, we included core electrons whereas Yang *et al*.^11^ applied frozen-core approximations. Both types of effects are of the order of 1 cm^−1^, i.e., are relevant for the spectral calculations^7^,^8^ and may be relevant for scattering calculations of the type presented here. One may mention here that, as shown in ref. 25, the post-CCSD(T) contribution for H_2_-CO is one of the largest among the 21 small weakly-bound complexes tested there.

Yang *et al*.^11^ used a quadruple-zeta quality basis set in an approach with explicitly-correlated terms (denoted by F12) which speeds up convergence in the basis set. If Yang *et al*. used an augmented F12 basis set, accuracy of their CCSD(T) interaction energies would have been similar to our Q5 extrapolated ones, but the lack of augmentation probably makes their CCSD(T) energies somewhat less accurate. Although we used only the TQ extrapolation for deformed monomers, the basis-set related difference between these two approaches amounts only to a fraction of a wave number and is anyway partly compensated by our hybrid procedure.

Yang *et al*.^11^ give only one value of the interaction energy, the “global minimum of the total potential” equal to −85.937 cm^−1^ (a computed value, not from the fit) for the collinear arrangement HH-CO, which in our notation corresponds to (*θ*_1_, *θ*_2_, *ϕ*) = (0°, 180°, 0°), and for *R* = 8.0 bohr. Then they say that this value is for intramonomer equilibrium coordinates *r* = 1.4011 bohr and *s* = 2.1359 bohr, which is unexpected since the global minimum of the 6-dimensional potential should be at different values of *r* and *s* than the equilibrium values of isolated monomers. Our vertical interaction energy (i.e., relative to the isolated monomers at the same separations as in the dimer) at this configuration is −90.263 cm^−1^ (obtained from our fit). This value is 4.326 cm^−1^ below the result of ref. 11. We should add that the minimum coordinates given in ref. 11 and quoted above are not exactly the same as the minimum coordinates on our surface; moreover, our C-O equilibrium separation is different from that given above. If we compute the interaction energy relative to the equilibria energies of our monomers, this value amounts to −88.461 cm^−1^. We presume that this energy should be compared with −85.937 cm^−1^ given given in ref. 11, which means that our energy is 2.524 cm^−1^ lower. Such difference is compatible with the differences in the theory level. In particular, the 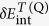 contribution to the interaction energy can be as large as −2.5 cm^−1^, see Table VI of ref. 8. Since our *ab initio* computed interaction energies were estimated in ref. 8 to have an uncertainty of 0.6 cm^−1^ near the minimum (or 0.8% of the interaction energy for all the investigated range in (*X*, *r*_*c*_, *s*_*c*_)), this comparison may indicate that the uncertainty of the potential of Yang *et al*.^11^ is about 4 times larger than that of our potential.

The actual minimum of the total potential energy surface *U*, defined according to Eq. (3) with the *V*_15_ interaction potential, is equal to −91.109 cm^−1^ and has been found for the collinear arrangement HH-CO with *R* = 7.898 bohr, *r* = 1.4021 bohr, and *s* = 2.1319 bohr. The intramolecular distances *r* and *s* are slightly changed in monomers compared to the isolated monomer equilibrium geometries, which are equal to 1.4011 and 2.1322 bohr for the 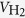 and *V*_CO_ monomer potentials, respectively. The corresponding vertical interaction energy amounts to −91.165 cm^−1^.

## Scattering Calculations

The scattering calculations were performed using an extended version of our DIDIMAT code which was first developed to study the H_2_-HF rigid-rotor collisions^26^ and used since then to study several other rigid-rotor diatom-diatom collisions like H_2_-CS^27^ and H_2_-CO^10^,^13^. The extended code does not use the rigid-rotor approximation and solves the complete close-coupling equations in the spaced-fixed frame by using a log-derivative propagator. General theory for diatom-diatom scattering in full dimensionality can be found in ref. 28. For the numerical calculations of the (isolated) diatom rovibrational energies and wave functions we employed the diatomic potentials presented in the previous section. A Discrete Variable Representation (DVR) of these functions was obtained by solving the diatomic equations using a basis of 150 complex exponential wave functions, as described, for example, by Colbert and Miller^29^. In the evaluation of the close-coupling potential matrix elements, these wave functions were integrated using a Gauss-Hermite quadrature grid of 10 points for each diatom (see SI for the numerical values), i.e., using a 100 point quadrature over *r* and *s*. Since the values of the 6D surface described earlier in this paper were needed only at these (*r*, *s*) points, this surface was refitted by direct expansions in products of spherical harmonics of all angular coordinates separately for each of the 100 values of (*r*, *s*) coordinates (resulting in 100 4D fits). At each value of *R*, the convergence of the angular expansion was checked and the size of the angular basis set was adapted accordingly, in order to save computer time and memory. See SI for details of the refit procedure. The calculations were performed for CO and H_2_ in their ground vibrational states as the influence of vibrationally excited states was found to be negligible at the investigated collision energies. As in the rigid-rotor calculations^10^,^13^, the highest rotational level of CO in the basis set was *j*_CO_ = 15, while two rotational levels of H_2_ were included for both the para (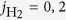) and ortho (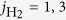) modifications. The maximum value of the total angular momentum *J* used in the calculations was *J* = 45. We have checked that such maximum values of the rotational quantum numbers were sufficient to converge the close-coupling calculations. The propagation was carried out to a maximum distance of 100 bohr for the lowest energy and the convergence was checked as a function of the propagator step size. Finally, a very fine grid of collision energies with 0.01 cm^−1^ increments was employed for the correct assessment of resonances.

In addition to full-dimensional scattering calculations, the rigid-rotor approximation was employed to assess the importance of full dimensionality. Three different 4D counterparts of the *V*_15_ surface were thus generated: the *V*_15_ surface averaged over the ground vibrations of the monomers, denoted as 〈*V*_15_〉_0_, the *V*_15_ surface with the bond lengths of H_2_ and CO fixed at their vibrationally averaged distances 〈*r*〉_0_ = 1.4487 bohr and 〈*s*〉_0_ = 2.1399 bohr, denoted as *V*_15_(〈*r*〉_0_), and the *V*_15_ surface with the bond lengths fixed at their equilibrium distances *r*_e_ = 1.4011 bohr and *s*_e_ = 2.1359 bohr, denoted as *V*_15_(*r*_e_). For these three 4D potential energy surfaces (PESs), the spherical harmonics refits were performed using the same angular basis set as for the 100 stretched configurations (see SI). In the rigid-rotor calculations, the rotational constants were fixed at their experimental values^30^ *B*_0_(CO) = 1.9225 cm^−1^ and *B*_0_(H_2_) = 59.322 cm^−1^. Note that rotational constants are not used in the 6D approach where the monomer rovibrational states are computed essentially exactly.

## Results

### Full-dimensional scattering calculations

Scattering cross-sections were computed for the range of collision energies covered by the crossed-beam experiment of Chefdeville *et al*.^10^. This experiment is one of very few which measured state-to-state cross sections with a relatively high accuracy. As mentioned earlier, the original analysis of the results contained an error due to inappropriate accounting for the mean interaction time and for the beam crossing median angle and its dispersion^31^.

**Figure 1:**
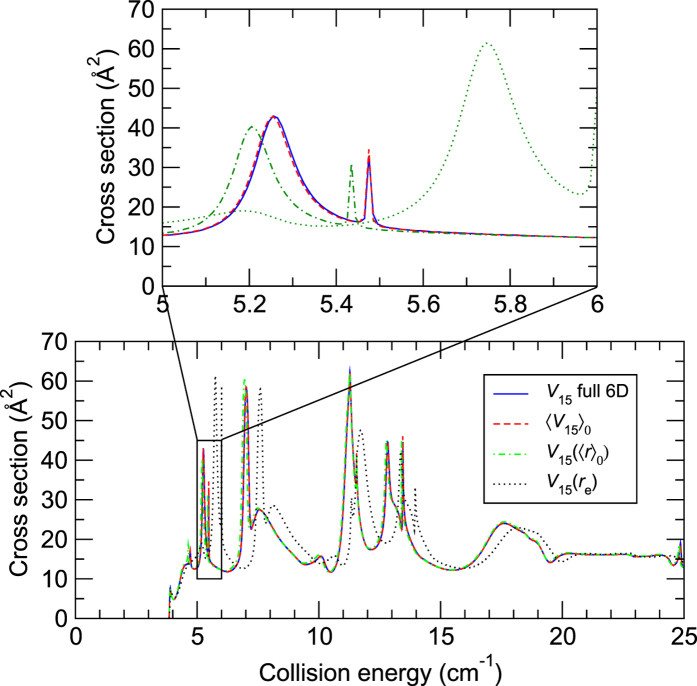
Cross sections for the CO excitation (*j*_CO_ = 0 → 1) due to para-H_2_(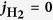) as functions of the collision energy. The top panel shows a zoom over collision energies between 5 and 6 cm^−1^. See text for details about the different levels of calculations.

**Figure 2:**
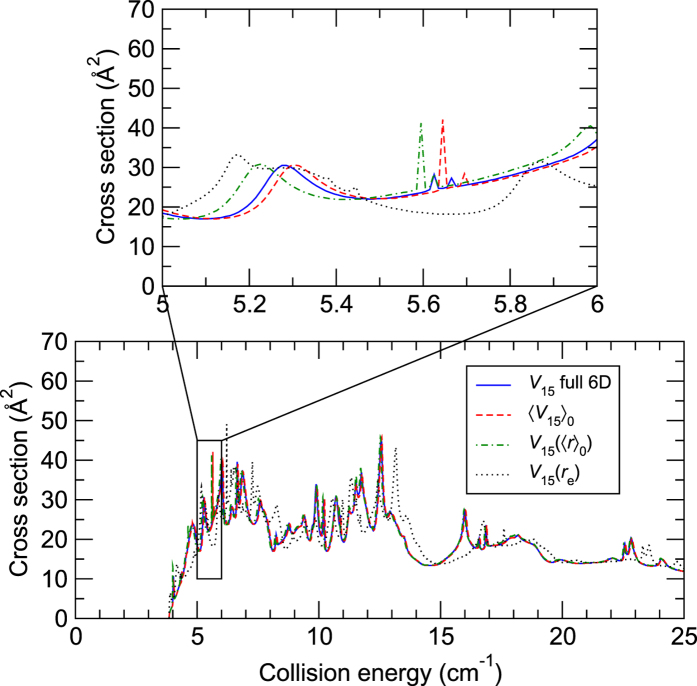
Cross sections for the CO excitation ( *j*_CO_ = 0 → 1) due to ortho-H_2_(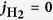) as functions of the collision energy. The top panel shows a zoom over collision energies between 5 and 6 cm^−1^. See text for details about the different levels of calculations.

The results in Figs 1 and 2 are shown for the *V*_15_ (6D) potential used in full-dimensional scattering and its three 4D approximations. The reported cross sections correspond to the fundamental rotational excitation of CO(*j*_CO_ = 0 → 1) by para-H_2_(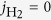) and ortho-H_2_(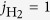). We note that the angular momentum 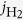 is conserved during these low-energy processes. From the bottom plots of Figs 1 and 2, we can notice that the full-dimensional calculations are in very good agreement with the 4D calculations performed using the 〈*V*_15_〉_0_ and *V*_15_(〈*r*〉_0_) potentials. In particular, the positions and intensities of the resonances are very well reproduced by these 4D calculations. In contrast, when the bond lengths are fixed at their equilibrium distances in the *V*_15_(*r*_e_) surface, the resonances are shifted by typically 0.5 cm^−1^ and their intensity is also modified by up to 50%. These relations are better illustrated in the top panels of Figs 1 and 2 which zoom in on the resonance region between 5 and 6 cm^−1^. Even after the zoom, the full-dimensional and 〈*V*_15_〉_0_ 4D results virtually overlap each other in the case of para-H_2_(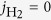). Clearly, the 4D 〈*V*_15_〉_0_ approximation gives extremely accurate description of this scattering process. In the case of ortho-H_2_(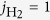), the two curves are shifted by about 0.02 cm^−1^. In order to get a better agreement, it is necessary to average *V*_15_ with the (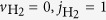) rovibrational wave function, as shown in the SI. In the case of the *V*_15_(〈*r*〉_0_) surface, the shifts are of the order 0.1 cm^−1^, also very small.

### Comparisons with crossed-beam experiments

**Figure 3:**
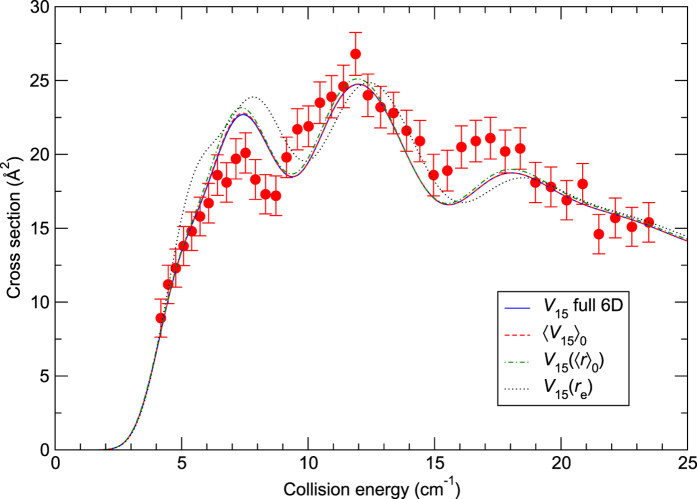
Cross sections for the CO excitation (*j*_CO_ = 0 → 1) due to para-H_2_(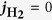) as functions of the collision energy. The experimental data (filled circles) are from ref. 10, where full details of the measurements can be found. The theoretical curves of Fig. 1 have been convolved with the experimental collision energy spread.

**Figure 4:**
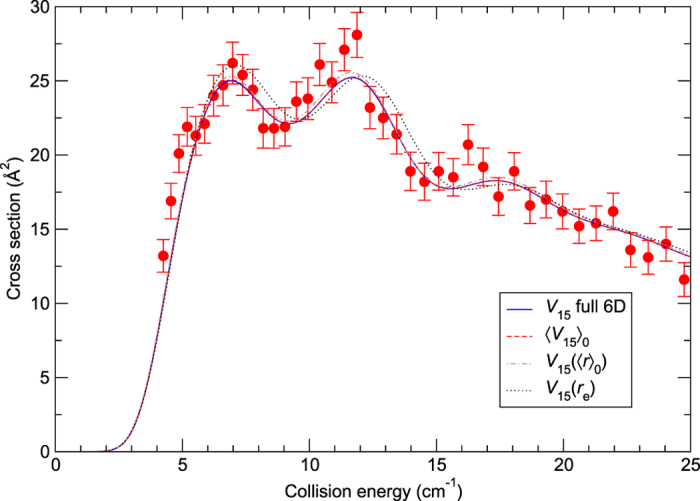
Cross sections for the CO excitation (*j*_CO_ = 0 → 1) due to normal-H_2_(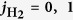) as functions of the collision energy. The experimental data (filled circles) are from ref. 10, where full details about the measurements can be found. The theoretical curves of Figs 1 and 2 have been convolved with the experimental collision energy spread and assuming 25% relative population of para-H_2_(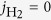) and 75% of ortho-H_2_(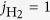).

As mentioned in the Introduction, the previous crossed-beam measurements of Chefdeville *et al*.^13^ were erroneous. They were corrected in ref. 10 where the data were reported for the rotational excitations *j*_CO_ = 0 → 1, *j*_CO_ = 0 → 2, and *j*_CO_ = 1 → 2. Cross sections for the excitation *j*_CO_ = 0 → 1 due to para-H_2_(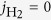) are plotted in Fig. 3. In this figure, the theoretical cross sections from Fig. 1 have been convolved with the experimental collision energy distribution (see SI for the details of the convolutions). It should be noted that the experimental data are measured in arbitrary units and the values reported here are scaled to theory as in Chefdeville *et al*.^10^. One can first notice that the agreement between theory and experiment is very good, especially in the threshold region, similar to that observed in ref. 10 where rigid-rotor calculations were performed using the 4D *V*_12_ surface. Second, the 6D calculations are in an excellent agreement with the 4D calculations, except for the case of the *V*_15_(*r*_e_) potential, as expected from Fig. 1, but even in the latter case the agreement with experiment is reasonable. Also, as expected from Fig. 1, results of the 4D calculations using 〈*V*_15_〉_0_ are hardly distinguishable from the 6D ones. Differences between these two approaches are orders of magnitude smaller than shown in Fig. 3 of Yang *et al*.^11^. Very similar relations can be observed in Fig. 4, where cross sections are reported for the excitation *j*_C*O*_ = 0 → 1 due to normal-H_2_ (25% para-H_2_(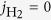) and 75% ortho-H_2_(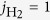)). Another set of experimental results are cross sections for the excitations *j*_CO_ = 0, 1 → 2 by para-H_2_(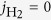). As shown in the SI, the agreement of the 6D calculations with these results is even closer than on the figures discussed above. This set, however, does not constitute a fully state-to-state comparison due to the contribution of both *j*_CO_ = 0 and *j*_CO_ = 1 to the scattering process. Our calculations show clearly that, in contrast to the conclusions of Yang *et al*.^11^, the predictions for the low-energy dynamics of the CO + H_2_ system *do not* require a full-dimensional approach to get agreement with the currently most accurate experimental results.

**Figure 5:**
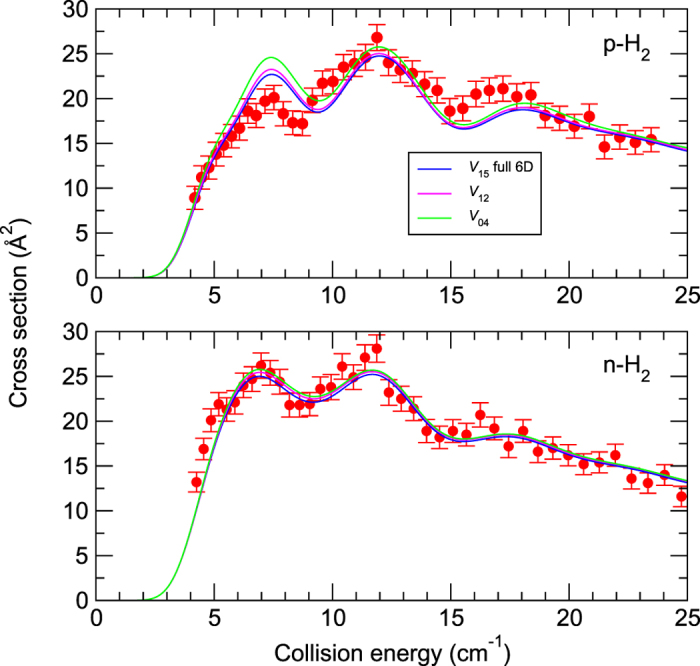
Cross sections for the CO excitation (*j*_CO_ = 0 → 1) due to para-H_2_ (top panel) and normal-H_2_ (bottom panel) as functions of the collision energy. The experimental data (filled circles) are from ref. 10. The theoretical curves correspond to three different surfaces: the present 6D *V*_15_ potential and the two 4D potentials, *V*_12_ and *V*_04_, from refs 4,8, respectively.

Another question concerning reliability of the theoretical predictions is the dependence on the accuracy of the potential energy surface at a given dimensionality, i.e., how close is the interaction energy predicted by a given surface to the exact interaction energy at a grid point. In order to illustrate the impact of the surface accuracy on the low-energy scattering, we report in Fig. 5 the 4D calculations performed with the previous *V*_04_ and *V*_12_ surfaces from refs 4,8, respectively, along with the present 6D treatment. We observe that the shapes of all (convolved) theoretical curves are very similar, with almost identical positions for the three resonance “peaks”. The cross sections tend to decrease with the increased accuracy of the potential. In particular, the agreement with the experiment improves significantly between *V*_04_ and *V*_15_ for para-H_2_ at low collision energies (below 10 cm^−1^). Still, the differences between the present 6D treatment and the 4D calculations performed on the *V*_12_ surface are negligible relative to the experimental error bars. The *V*_12_ surface is a 〈*V*〉_0_-type surface, obtained at the same level of *ab initio* theory as the present potential, but with less dense sampling of the configuration space. Clearly, our increase in the number of grid points had a very small effect on the cross sections. The *V*_04_ surface was partly averaged, only over the H_2_ vibrations, whereas the CO separation was held at 〈*s*〉_0_. This surface was also less accurate than *V*_15_ or *V*_12_ since a lower level of theory was used. The differences between the former and the two latter surfaces are of the order of 1 cm^−1^ in the minimum region. Thus, about 1% relative errors in the interaction energies lead to only a minor (if any, since the *V*_04_ results are closer to experiment in some ranges of collision energy) worsening of agreement with experiment.

The fairly large discrepancies between the calculations of Yang *et al*.^11^ and the correct experiments (or our results) may indicate problems related to the asymptotics of their potential. As discussed earlier, their potential and *V*_15_ differ likely by 2–3 cm^−1^ in the minimum region, insufficient to explain the discrepancies. However, the asymptotics effect may be large in low-energy scattering. Thus, the extension of the methodology to all degrees of freedom in the work of Yang *et al*.^11^ resulted in a deterioration of the quality of predictions probably due to trade-offs concerning the accuracy of the potential surface (the level of theory, the size of the basis set, and the frozen-core approximation), and due to the asymptotic form of the fit.

## Discussion

The present study of the H_2_-CO inelastic scattering has been carried out to investigate the effects of full-dimensionality at low scattering energies, less than 25 cm^−1^, close to the threshold for rotational excitations. A new high-level 6D potential energy surface was combined with nearly-exact full-dimensional close-coupling scattering calculations to provide the most detailed results to date in this cold regime. We focused on the fundamental rotational excitation of CO (*j*_CO_ = 0 → 1) due to para- and normal-H_2_, for which the most accurate experimental results are available. The accuracy of the 6D surface was demonstrated by the very good agreement between the predicted and experimental values of state-to-state cross sections. The 4D scattering calculations based on the 〈*V*_15_〉_0_ surface (*V*_15_ averaged over monomer vibrations) give results almost indistinguishable from the 6D ones, in agreement with findings of ref. 9. The 4D treatment based on the *V*_15_(〈*r*〉_0_) surface (*V*_15_ computed at the vibrationally averaged intramonomer separations) is also very close to the experiment and confirms reliability of the rigid-rotor approximation. The accuracy of predictions drops fairly substantially (e.g., the shifts of resonance positions are about 5 times larger) if the 4D potential is computed at equilibrium separations.

Yang *et al*.^11^ have also computed cross sections at high collision energy and for the vibrational deexcitation of CO from *v*_CO_ = 1 state. We have performed such calculations as well, but do not report detailed results since none of the two cases allows one to make conclusions on the importance of the 6D treatment versus the 4D one. In the high-energy case, Yang *et al*.^11^ already found that the two treatments lead to essentially the same agreement with the results for rotational excitations of CO measured in a crossed-beam experiment at collision energies near 1000 cm^−1^ in ref. 12, and our findings are the same. In the case of vibrational deexcitation, one has to use a surface with at least the C-O distance dependence, so only the effects of including or not the H-H separation can be investigated. When restricting CO to its ground rotational state (*v*_CO_ = 1, *j*_CO_ = 0), our rate coefficients for this process agree with the experiment of ref. 32 to within a factor of 2 above 200 K. At lower temperature, however, our results are significantly larger than experiment and also larger than the results of Yang *et al*. We note, however, that the experimental results were not fully state-resolved but rather correspond to distributions (sometimes unknown) of the rotational CO and H_2_ states. Thus, these results are much less certain than the crossed-beam data of ref. 10 and less appropriate for validating various levels of theory. A detailed report on our calculations will be published separately.

In conclusion, the current results show that full-dimensionality is not required to predict low-energy (from the threshold to 25 cm^−1^) scattering cross-sections that are sufficiently accurate for astrophysical applications and competitive in accuracy with recent experimental results. These findings have important consequences for astrophysical applications since inelastic state-to-state rate coefficients are necessary for a variety of molecules at very low temperatures^33^. In particular, complex organic molecules such as methyl formate (HCOOCH_3_) are now observed routinely in cold pre-stellar cores where the kinetic temperature can be as low as a few kelvin^34^. For such species with many vibrational modes, full-dimensional treatments are currently not possible and even rigid-rotor calculations are challenging^35^. The present work demonstrates that current reduced-dimensional calculations can reach such high accuracy that the corresponding collisional rate coefficients are not a limiting factor in the interpretation of astronomical molecular spectra.

## Additional Information

**How to cite this article**: Faure, A. *et al*. On the importance of full-dimensionality in low-energy molecular scattering calculations. *Sci. Rep.* **6**, 28449; doi: 10.1038/srep28449 (2016).

## Supplementary Material

Supplementary Information

## Figures and Tables

**Table 1:** Comparison of the present methodology of developing potential energy surface with that of ref. 11.

	Ref. 11	Present
range of *R*	4–18 bohr	4.5–20 bohr
range of *r*	1.01–1.81 bohr	0.95–2.05 bohr
range of *s*	1.74–2.54 bohr	1.90–2.45 bohr
energy cutoff	10,000 cm^−1^	1,000 cm^−1^
number of grid points	398,218	37,894
fitting function	sum of products of Morse-type variables	sum of powers times exponentials of *R* and of inverse powers of *R* with coefficients dependent on remaining coordinates
RMSE	14.22 cm^−1^	0.63 cm^−1^
method	CCSD(T)-F12 frozen core	up to CCSDT(Q) effectively all electrons
basis set	cc-pVQZ-F12	up to aug-cc-pV5Z

## References

[b1] Omont A. Molecules in galaxies. Rep. Prog. Phys. 70, 1099–1176 (2007).

[b2] Green S. & Thaddeus P. Rotational excitation of CO by collisions with He, H, and H_2_ under conditions in interstellar clouds. Astrophys. J. 205, 766–785 (1976).

[b3] Jankowski P. & Szalewicz K. *Ab initio* potential energy surface and infrared spectra of H_2_-CO and D_2_-CO van der Waals complexes. J. Chem. Phys. 108, 3554–3565 (1998).

[b4] Jankowski P. & Szalewicz K. A new *ab initio* interaction energy surface and high-resolution spectra of the H_2_-CO van der Waals complex. J. Chem. Phys. 123, 104301 (2005). 16178591 10.1063/1.2008216

[b5] Kobayashi R., Amos R. D., Reid J. P., Quiney H. M. & Simpson C. J. S. M. Coupled cluster *ab initio* potential energy surface for CO…He and CO…H_2_. Mol. Phys. 98, 1995–2005 (2000).

[b6] Flower D. R. Rate coefficients for the rovibrational excitation of CO by H_2_ and He. Mon. Not. Roy. Astron. Soc. 425, 1350–1356 (2012).

[b7] Jankowski P., McKellar A. R. W. & Szalewicz K. Theory untangles the high-resolution infrared spectrum of the *ortho*H_2_-CO van der Waals complex. Science 336, 1147–1150 (2012). 22654055 10.1126/science.1221000

[b8] Jankowski P. . A comprehensive experimental and theoretical study of H_2_-CO spectra. J. Chem. Phys. 138, 084307 (2013). 23464151 10.1063/1.4791712

[b9] Jeziorska M., Jankowski P., Szalewicz K. & Jeziorski B. On the optimal choice of monomer geometry in calculations of intermolecular interaction energies: Rovibrational spectrum of Ar-HF from two- and three-dimensional potentials. J. Chem. Phys. 113, 2957–2968 (2000).

[b10] Chefdeville S. . Experimental and theoretical analysis of low-energy CO + H_2_ inelastic collisions. Astrophys. J. Lett. 799, L9 (2015).

[b11] Yang B. . Quantum dynamics of CO-H_2_ in full dimensionality. Nature Comm. 6, 6629 (2015). 10.1038/ncomms7629 25800802

[b12] Antonova S., Tsakotellis A. P., Lin A. & McBane G. C. State-to-state rotational excitation of CO by H_2_ near 1000 cm^−1^ collision energy. J. Chem. Phys. 112, 554–559 (2000).

[b13] Chefdeville S. . Appearance of low energy resonances in CO-para-H_2_ inelastic collisions. Phys. Rev. Lett. 109, 023201 (2012). 23030157 10.1103/PhysRevLett.109.023201

[b14] Costes M. & Naulin C. Observation of quantum dynamical resonances in near cold inelastic collisions of astrophysical molecules. Chem. Sci. 7, 2462–2469 (2016). 10.1039/c5sc04557f PMC5477044 28660016

[b15] Kendall R. A., Dunning T. H. Jr. & Harrison R. J. Electron-affinities of the 1^st^-row atoms revisited–systematic basis-sets and wave-functions. J. Chem. Phys. 96, 6796–6806 (1992).

[b16] Leforestier C., Szalewicz K. & van der Avoird A. Spectra of water dimer from a new *ab initio* potential with flexible monomers. J. Phys. Chem. 137, 014305 (2012). 10.1063/1.4722338 22779646

[b17] Garberoglio G., Jankowski P., Szalewicz K. & Harvey A. H. Second virial coefficients of H_2_ and its isotopologues from six-dimensional potential. J. Chem. Phys. 137, 154308 (2012). 23083166 10.1063/1.4757565

[b18] Jankowski P. . Ab initio water pair potential with flexible monomers. J. Phys. Chem. A 119, 2940–2964 (2015). 25687650 10.1021/jp512847z

[b19] Szalewicz K., Murdachaew G., Bukowski R., Akin-Ojo O. & Leforestier C. Spectra of water dimer from *ab initio* calculations. In Maroulis G. & Simos T. (eds.) Lecture Series on Computer and Computational Science: International Conference on Computational Methods in Science and Engineering (ICCMSE 2006), vol. 6, 482–491 (Brill Academic Publishers, Leiden, 2006).

[b20] Bukowski R. . Intermolecular potential of carbon dioxide dimer from symmetry-adapted perturbation theory. J. Chem. Phys. 110, 3785–3803 (1999).

[b21] Jeziorski B., Moszynski R. & Szalewicz K. Perturbation theory approach to intermolecular potential energy surfaces of van der Waals complexes. Chem. Rev. 94, 1887–1930 (1994).

[b22] Pachucki K. Born-Oppenheimer potential for H_2_. Phys. Rev. A 82, 032509 (2010).

[b23] SongL., van der AvoirdA. & GroenenboomG. C. Three-dimensional ab initio potential energy surface for HCO( 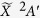 ). J. Phys. Chem. A 117, 7571–7579 (2013).2359713310.1021/jp402470b

[b24] Le Floch A. Revised molecular constants for the ground state of CO. Mol. Phys. 72, 133–144 (1991).

[b25] Smith D. G. A., Jankowski P., Slawik M., Witek H. A. & Patkowski K. Basis set convergence of the post-CCSD(T) contribution to noncovalent interaction energies. J. Chem. Theory Comput. 10, 3140–3150 (2014). 26588285 10.1021/ct500347q

[b26] Guillon G., Stoecklin T., Voronin A. & Halvick P. Rotational relaxation of HF by collision with ortho- and para-H_2_ molecules. J. Chem. Phys. 129, 104308 (2008). 19044914 10.1063/1.2975194

[b27] Denis-Alpizar O., Stoecklin T., Halvick P. & Dubernet M.-L. Rotational relaxation of CS by collision with ortho- and para-H_2_ molecules. J. Chem. Phys. 139, 204304 (2013). 24289351 10.1063/1.4832385

[b28] Alexander M. H. & DePristo A. E. Symmetry considerations in the quantum treatment of collisions between two diatomic molecules. J. Chem. Phys. 66, 2166–2172 (1977).

[b29] Colbert D. T. & Miller W. H. A novel discrete variable representation for quantum mechanical reactive scattering via the S-matrix Kohn method. J. Chem. Phys. 96, 1982–1991 (1992).

[b30] Huber K. P. & Herzberg G. Molecular Spectra and Molecular Structure. IV. Constants of diatomic molecules (Van Nostrand Reinhold, New York, 1979).

[b31] Naulin C. & Costes M. Experimental search for scattering resonances in near cold molecular collisions. Int. Rev. Phys. Chem. 33, 427–446 (2014).

[b32] Andrews A. J. & Simpson C. J. S. M. Vibrational deactivation of CO by n-H_2_, by p-H_2_ and by HD measured down to 77 K using laser fluorescence. Chem. Phys. Lett. 41, 565–569 (1976).

[b33] Roueff E. & Lique F. Molecular excitation in the interstellar medium: recent advances in collisional, radiative, and chemical processes. Chem. Rev. 113, 8906–8938 (2013). 24131323 10.1021/cr400145a

[b34] Bacmann A., Taquet V., Faure A., Kahane C. & Ceccarelli C. Detection of complex organic molecules in a prestellar core: a new challenge for astrochemical models. Astron. Astrophys. 541, L12 (2012).

[b35] Faure A., Remijan A. J., Szalewicz K. & Wiesenfeld L. Weak maser emission of methyl formate toward Sagittarius B2(N) in the Green Bank Telescope PRIMOS survey. Astrophys. J. 783, 72 (2014).

