# Supplementary material for: On the importance of full-dimensionality in low-energy molecular scattering calculations
Source: Sci Rep. 2016 Jun 23;6:28449. doi: 10.1038/srep28449 (PMC4917847; doi:10.1038/srep28449)
Supplement: Supplementary Information [file srep28449-s1.pdf]

Supplementary Information for: On the importance of full-dimensionality in low-energy molecular scattering calculations

Alexandre Faure,[1,2] Piotr Jankowski,[3] Thierry Stoecklin,[4] and Krzysztof Szalewicz[5]

[1] *Université de Grenoble Alpes, IPAG, F-38000 Grenoble, France*

[2] *CNRS, IPAG, 38000 Grenoble, France*

[3] *Faculty of Chemistry, Nicolaus Copernicus University in Torun, Gagarina 7, PL-87-100 Torun, Poland*

[4] *Institut des Sciences Moleculaires, Universite de Bordeaux, F-33405 Talence, France*

[5] *Department of Physics and Astronomy, University of Delaware, Newark, DE 19716*

(Dated: May 20, 2016)

I. GRID

The six-dimensional grid in the $\boldsymbol{XY} = (R, \theta_1, \theta_2, \phi, r, s)$ coordinates was obtained as a direct product of the intermolecular $\boldsymbol{X} = (R, \theta_1, \theta_2, \phi)$ and intramolecular $\boldsymbol{Y} = (r, s)$ grids. The parameter R denotes the separation between the centers of mass (COM) of H_2 and CO. To define the angles, let us choose the z axis going through these COMs and oriented from H_2 to CO. The parameter ϕ denotes the dihedral angle between half planes going through this axis and one of the H atoms (denoted as H1) and the C atom. The angles of the vectors formed by the COMs and these two atoms with the z axis are denoted as θ_1 and θ_2, respectively (with zero values for the COM-atom vectors colinear with the axis). The coordinates r and s are the H-H and C-O distances, respectively.

The intermolecular grid is similar to that used in Ref. 1, i.e., it is a direct product of grids in R and $(\theta_1, \theta_2, \phi)$. The following 18 values of R (in bohr) have been used: 5.0, 5.25, 5.5, 5.75, 6.0, 6.5, 7.0, 7.5, 8.0, 8.5, 9.0, 10.0, 11.0, 12.0, 13.5, 15.0, 17.5, 20.0. This radial grid has been combined with the angular one, defined as in Ref. 2 by a set of 33 unique combinations of the following angles: 0°, 45°, 90°, 135° for θ_1; 0°, 45°, 90°, 135°, 180° for θ_2; 0°, 45°, 90° for ϕ. For four intermolecular distances, 7.0, 8.0, 10.0, and 15.0 bohr, the angular grid was made twice as dense in each of the θ_1, θ_2, and ϕ coordinates, which resulted in 208 additional angular configurations. Thus, the total number of intermolecular grid points resulting from the regular grid defined above is equal to $18{\cdot}33{+}4{\cdot}208 = 1{,}426$.

The grid in the intramonomer coordinates $\boldsymbol{Y} = (r, s)$ was constructed as a direct product of 5 points in r: 0.95, 1.2, 1.4, 1.67, 2.05 bohr, and 5 points in s: 1.9, 1.99, 2.13, 2.30, 2.45 bohr. One more grid point was $(r_c, s_c) = (1.474, 2.165)$ bohr. These values are not any of the vibrationally averaged distances but are chosen to lie between the values averaged in the ground and first excited states. This choice was made in Refs. 1 and 3 to perform Taylor expansions of the surface around these points. Thus, the two-dimensional grid in the $\boldsymbol{Y}$ coordinates is comprised of 26 points. The direct product of the inter- and intramolecular grids results in 37,076 points of the total regular grid in the $\boldsymbol{XY}$ coordinates.

The use of very large interaction energies in the data set leads to some bias when fitting the potential since the errors related to such points are large and difficult to control with energy-based weights. Therefore, we decided to include only interaction energies below 1000 cm^{-1} (this resulted in not using 1,922 of the computed energies). On the other hand, our

goal was to cover all regions of the coordinate space with interaction energies below this threshold. Therefore, for the smallest distance from the regular grid, $R = 5.0$ bohr, we identified orientations with interaction energy values smaller than 1000 cm^{-1}. For such angular orientations, the interaction energy was calculated for R equal to 4.75 and 4.5 bohr, combined with all intramonomer grid points in the $\boldsymbol{Y}$ coordinates. This resulted in 832 additional grid points and the total number of grid points in the six-dimensional space defined by the $\boldsymbol{XY}$ coordinates was thus increased to 37,908. However, for 14 of these points, all corresponding to small values of R and the repulsive part of the potential, *ab initio* calculations did not converge. Thus, the final, six-dimensional grid comsists of 37,894 points (but, as mentioned, 1,922 of these points were not used in the fit).

For all of these 37,894 points, the interaction energy denoted further as $E_{\rm int,B}$, where "B" in the subscript denotes the 'base' level of theory and basis set, was calculated as follows. The Hartree-Fock interaction energy, $E_{\rm int}^{\rm HF}[{\rm Q}]$, was computed using the aug-cc-pVQZ basis set[4]. The correlation part, $\delta E_{\rm int}^{\rm CCSD(T)}[{\rm TQ}]$, was computed using the coupled-cluster method with single, double, and noniterative triple excitations [CCSD(T)] in the aug-cc-pVXZ, $X =$ T and Q, basis sets[4]. These results were used to perform $1/X^3$ (Ref. 5) extrapolation to the complete basis set (CBS) limit. The extrapolated result is denoted as $\delta E_{\rm int}^{\rm CCSD(T)}[{\rm TQ}]$. Thus, the interaction energy is

$$E_{\rm int,B} = E_{\rm int}^{\rm HF}[{\rm Q}] + \delta E_{\rm int}^{\rm CCSD(T)}[{\rm TQ}].$$

At the grid points with intramonomer coordinates $(r, s) = (r_c, s_c)$, we have performed calculations at a higher level of theory: CCSD(T) calculations in the aug-cc-pV5Z basis set and CCSDT(Q) calculations in aug-cc-pVDZ basis set, where here T stands for iterated triple excitations and (Q) stands for noniterated quadruple ones. The latter calculations applied the frozen-core approximation whereas all other calculations were done with all electrons. This defines two corrections:

$$\Delta E_{\rm int}[{\rm TQ;Q5}] = E_{\rm int}^{\rm HF}[5] + \delta E_{\rm int}^{\rm CCSD(T)}[{\rm Q5}] - E_{\rm int,B},$$

and

$$\delta E_{\rm int}^{\rm T(Q)}[{\rm D}] = E_{\rm int}^{\rm CCSDT(Q)}[{\rm D}] - E_{\rm int}^{\rm CCSD(T)}[{\rm D}].$$

Thus, the interaction energy at the high-level of theory is defined as

$$E_{\rm int,H} = E_{\rm int,B} + \Delta E_{\rm int}[{\rm TQ;Q5}] + \delta E_{\rm int}^{\rm T(Q)}[{\rm D}]. \tag{1}$$

To combine the two sets of results, we have employed the idea of the hybrid potential introduced in Refs. 6–8 (also indirectly in Refs. 1 and 3). If, for an arbitrary complex, one knows a full-dimensional surface $V_{\rm full}$, obtained at a moderate level, and a rigid-monomer surface $V_{\rm rigid}$, obtained at high level, the hybrid surface can be defined as

$$V_{\rm hyb}(\boldsymbol{d}) = V_{\rm full}(\boldsymbol{d}) + (V_{\rm rigid}(\boldsymbol{u}) - V_{\rm full}(\boldsymbol{u})) \equiv V_{\rm rigid}(\boldsymbol{u}) + (V_{\rm full}(\boldsymbol{d}) - V_{\rm full}(\boldsymbol{u})), \quad (2)$$

where $\boldsymbol{d} = (R, \theta_1, \theta_2, \phi, r, s)$ and $\boldsymbol{u} = (R, \theta_1, \theta_2, \phi, r_c, s_c)$ denote geometries with distorted and the corresponding undistorted (i.e., rigid-geometry) monomers, respectively. For nonlinear monomers, the definition depends on the embedding of monomers in the dimer[8]. The first grouping in Eq. (2) can be interpreted as adding a correction for the extended theory level computed only at rigid geometries to the lower-level full-dimensional potential. The second grouping amounts to adding a monomer distortion correction computed at a lower level of theory to a more accurate rigid-monomer potential. In the previous work, the "full" and "rigid" potentials on the right-hand side of Eq. (2) were individually fitted. In contrast, we applied Eq. (2) to individual grid points

$$E_{\rm int,hyb}(\boldsymbol{X}, r, s) = E_{\rm int,B}(\boldsymbol{X}, r, s) + (E_{\rm int,H}(\boldsymbol{X}, r_c, s_c) - E_{\rm int,B}(\boldsymbol{X}, r_c, s_c)). \quad (3)$$

For grid points with $5.0 \le R < 5.5$ bohr, we replaced Eq. (1) by $\tilde{E}_{\rm int,H} = E_{\rm int,B} + \delta E_{\rm int}^{\rm T(Q)}[{\rm D}]$ since for some angular orientations there were problems with converging the interaction energy calculations in the aug-cc-pV5Z basis set. For grid points with $R < 5.0$ bohr, we used only $E_{\rm int,B}$, as these values are in a highly repulsive region and do not need to be very accurate. The set of interactions energies defined by Eq. (3) was used to fit a 6-dimensional potential.

To check how the hybrid approximation works, we computed $E_{\rm int,H}$ for a set of deformed monomer geometries: $\boldsymbol{XY} = (8.0, 0°, 180°, 0°, r, s)$ and $\boldsymbol{XY} = (7.0, 0°, 0°, 0°, r, s)$ for all 25 (r, s) values in each case. The results of Table I show that for (r, s) close to (r_c, s_c), the approximation works very well, i.e., the hybrid results are closer to the $E_{\rm int,H}$ than the $E_{\rm int,B}$ values are. However, for (r, s) farthest from (r_c, s_c), in particular for extensions of the C-O bond at $R = 7$ bohr, the approximation is sometimes poor and in 22% of cases the hybrid results are less accurate than the $E_{\rm int,B}$ values. One may try to devise a scheme of gradually phasing out the high-level correction for more distorted monomers, but we could not find any sensible approach. The hybrid Ansatz of Eq. (2) was shown to work well for the water

dimer as the spectra of this system computed in Ref. 6 agreed overall better with experiment than the spectra calculated with the base potentials in Ref. 9.

II. ANALYTIC FIT

The analytic representation of the 6-dimensional potential energy surface is a generalization of the 4-dimensional surfaces of the type developed in Refs. 1, 2, 10, and 11 in the sense that the parameters of the intermolecular part are expanded in polynomials of r and s. The surfaces of this type consist of short-range and long-range (asymptotic) components $V_{\rm sh}$ and $V_{\rm as}$, respectively, so that the total potential is

$$V(R,\theta_1,\theta_2,\phi,r,s) = V_{\rm sh}(R,\theta_1,\theta_2,\phi,r,s) + V_{\rm as}(R,\theta_1,\theta_2,\phi,r,s). \tag{4}$$

The short-range part is defined in a similar way as in Ref. 11, except that the fitting function depends not only on the intermolecular coordinates, but also on the intramolecular ones r and s, i.e.,

$$V_{\rm sh}(R,\theta_1,\theta_2,\phi,r,s) = \left[\sum_{i=0}^{3} R^i C_i(\theta_1,\theta_2,\phi,r,s)\right] {\rm e}^{D(\theta_1,\theta_2,\phi,r,s)-B(\theta_1,\theta_2,\phi,r,s)R}. \tag{5}$$

The angular factors in Eq. (5) are defined as

$$C_i(\theta_1,\theta_2,\phi,r,s) = \sum_{l_1,l_2,l}\sum_{j=1}^{10} g_i^{l_1l_2l,j} A_{l_1l_2l}(\theta_1,\theta_2,\phi)\, w_j(r,s), \tag{6}$$

$$B(\theta_1,\theta_2,\phi,r,s) = \sum_{l_1,l_2,l}\sum_{j=1}^{6} b^{l_1l_2l,j} A_{l_1l_2l}(\theta_1,\theta_2,\phi)\, w_j(r,s), \tag{7}$$

and D is analogous to B. The polynomials $w_j(r,s)$ are defined in Table II. The definitions of the angular functions $A_{l_1l_2l}(\theta_1,\theta_2,\phi)$ can be found in Ref. 11. The sum in Eq. (6) runs over the (l_1,l_2,l) indices in the set of 25 arrangements, whereas the sum in Eq. (7) runs over 8 arrangements; both sets are listed in Ref. 1. Thus, there are 1000 coefficients $g_i^{l_1l_2l,j}$, 48 $b^{l_1l_2l,j}$, and 48 $d^{l_1l_2l,j}$.

The $V_{\rm as}$ component is given by

$$V_{\rm as}(R,\theta_1,\theta_2,\phi,r,s) = \sum_{n=4}^{12}\sum_{l_1,l_2,l} f_n[B(\theta_1,\theta_2,\phi,r,s)R]\frac{C_n^{l_1l_2l}}{R^n}\Gamma_n(\theta_1,\theta_2,\phi,r,s)\, A_{l_1l_2l}(\theta_1,\theta_2,\phi), \tag{8}$$

where f_n are the Tang-Toennies damping functions[12] and B is the same as in Eq. (5). This form was introduced in Ref. 11 except for the scaling factor $\Gamma_n(\theta_1, \theta_2, \phi, r, s)$. A constant scaling factor was used in the 4-dimensional fit of Ref. 1, but we found that such form was insufficient for the 6-dimensional fit. The $C_n^{l_1 l_2 l}$ coefficients contain information about the asymptotic electrostatic, induction, and dispersion components of the interaction energy. The index n in Eq. (8) runs from 4 to 12, where the $n = 4$ term originates from the quadrupole-dipole electrostatic interaction of the H_2 and CO molecules. These coefficients were *ab initio* computed in Ref. 11 from monomers' multipole moments and polarizabilities (static and dynamic) at the level consistent with the level of symmetry-adapted perturbation theory (SAPT)[13] used in Ref. 10. This level of theory is different from that of the present work and also our basis sets are much larger than used in Ref. 11. Since the asymptotics of the CCSDT(Q) method is unknown, we have decided to take the numerical values of $C_n^{l_1 l_2 l}$ from Ref. 11 and introduce factors Γ_n of the form

$$\Gamma_n(\theta_1, \theta_2, \phi, r, s) = \sum_{k_1, k_2, k} \sum_{j=1}^{6} \gamma_n^{k_1 k_2 k, j} A_{k_1 k_2 k}(\theta_1, \theta_2, \phi)\, w_j(r, s), \;\; n = 4, \ldots, 10, \tag{9}$$

where the first sum runs over 4 sets of $(k_1 k_2 k)$: $(0,0,0), (0,1,1), (0,2,2), (2,0,2)$. The values of 168 $\gamma_n^{k_1 k_2 k, j}$ parameters, $(7 \times 4 \times 6)$, were obtained by least squares fitting of V_{as} without damping functions, i.e., with $f_n = 1$, to the 16,822 long-range energies $E_{\text{int,hyb}}(\boldsymbol{X}, r, s)$ with $R \geq 10$ bohr. The Γ_n parameters for $n > 10$ were set to 1. In the least squares fitting of V_{as}, the interaction energies were weighted by a factor $w_a = \left(\frac{R}{R_w}\right)^8$, where $R_w = 10$ bohr. One may argue that a more consistent way of scaling would be to introduce coefficients $\Gamma_n^{l_1 l_2 l}(r, s)$, i.e., to scale each $C_n^{l_1 l_2 l}$ by a factor depending on intramonomer coordinates. However, since the number of coefficients $C_n^{l_1 l_2 l}$ with $n \leq 10$ is 80, this would give 6×80, too many linear parameters.

The final potential was fitted to the 27,132 values of $E_{\text{int,hyb}}(\boldsymbol{X}, r, s)$, using the functional form of Eq. (4) with the asymptotic part V_{as} frozen, except for the parameters $b^{l_1 l_2 l, j}$. Out of the total number of 37,894 computed interaction energies, we rejected 1,922 values larger than 1000 cm^{-1} and 8,840 values with $R > 12$ bohr. The high-energy values are unimportant for the intended applications of our fit and the values at $R > 12$ bohr are already well reproduced by the asymptotic part of the fit.

We used the following weights in the fit:

$$w_t = w_E w_r w_s \tag{10}$$

$$w_E = \begin{cases} \left(\frac{E_w}{E}\right)^2 & \text{for } E > E_w \\ e^{-3(E-E_w)/2E_w} & \text{for } E \le E_w \end{cases}, \tag{11}$$

$$w_r = \frac{1}{1+\left(\frac{r-r_w}{0.6}\right)^2}\ ,\ w_s = \frac{1}{1+\left(\frac{s-s_w}{0.3}\right)^2}, \tag{12}$$

where $E_w = 175$ cm^{-1}, $r_w = 1.45$ bohr, and $s_w = 2.14$ bohr. The values of the weighting factors w_r and w_s are smallest for the values of r and s which differ most from $r_w \approx r_c$ and $s_w \approx s_c$, respectively. This means that the energies calculated with the highest accuracy, i.e., with $r \approx r_c$ and $s \approx s_c$, are weighted most in the fit.

The accuracy of the fit can be characterized by the root-mean-square error (RMSE), which is equal to 0.629 cm^{-1} for all 27,132 grid points used in the final fit. For the 22,668 negative energies, i.e., belonging to the interaction potential well, the RMSE amounts to 0.164 cm^{-1}, whereas for the 4,464 positive energies (smaller than our threshold of 1000 cm^{-1}), the RMSE is equal to 1.505 cm^{-1}.

To further check the quality of our fit, we calculated the $E_{\text{int,hyb}}$ interaction energies at 200 randomly chosen points, applying the following restrictions: $R \in [5.5, 10.0]$, $r \in [0.95, 2.05]$, and $s \in [1.90, 2.45]$ bohr. The angles were generated within their full ranges. Four energies from this random set were larger than 1000 cm^{-1} and were discarded. For the 153 points with negative energies, the RMSE of the V_{15} potential with respect to the *ab initio* energies was equal to 0.42 cm^{-1}. The corresponding RMSE for the negative energies obtained on the grid used in the fitting procedure, but restricted to the same ranges of R, r, and s, was equal to 0.17 cm^{-1}. For the 43 remaining positive energies, the RMSE amounted to 5.45 cm^{-1}, whereas for the corresponding energies used in the fit, it is equal to 1.37 cm^{-1}. Thus, the increase of RMSE was a factor of 2.5 and 4, respectively. While this increase was relatively substantial, the RMSE for negative energies is still smaller than the estimated uncertainty of our *ab initio* interaction energies, amounting to 0.6 cm^{-1}. Thus, we concluded that calculations of additional interaction energies was not warranted.

III. SPHERICAL HARMONICS FIT

Since quantum scattering calculations require an expansion of the angular dependence of the potential energy surface into products of spherical harmonics, the potential described in Sec. II had to be refitted. Due to the way the scattering calculations are done, the refit was

made for a set of fixed values of (r, s), i.e., it consisted of a set of 4D fits. The fit of Eq. (4) was employed to generate interaction energies on a very dense grid of 7,500,000 geometries in R, $(\theta_1, \theta_2, \phi)$, and (r, s). The following 25 values of R (in bohr) have been used: 4.00, 4.25, 4.50, 4.75, 5.00, 5.25, 5.50, 5.75, 6.00, 6.25, 6.50, 6.75, 7.00, 7.25, 7.50, 8.00, 8.50, 9.00, 9.50, 10.00, 11.00, 12.00, 13.00, 14.00 and 15.00. This radial grid has been combined with 3000 random angular geometries $(\theta_1, \theta_2, \phi)$. The grid in the intramonomer coordinates (r, s) has been obtained as a direct product of Gauss-Hermite quadrature points: 10 points in r (0.60, 0.81, 0.99, 1.16, 1.32, 1.48, 1.64, 1.81, 1.99, 2.2) bohr and 10 points in s (1.82, 1.90, 1.97, 2.04, 2.10, 2.16, 2.23, 2.29, 2.36 and 2.44) bohr. We note that some of these points lie outside the ranges of our *ab initio* data. We have checked, however, that the fit is still stable at the extreme values, and, in any case, the contribution of these points to the quadrature is almost negligible. A set of 100 4D grids with 75,000 energy points each were thus produced, and each such set was fitted by a 4D analytical expansion of the form

$$V(R, \theta_1, \theta_2, \phi) = \sum_{l_1 l_2 l} v_{l_1 l_2 l}(R) s_{l_1 l_2 l}(\theta_1, \theta_2, \phi), \tag{13}$$

where the basis functions $s_{l_1 l_2 l}$ are products of associate Legendre polynomials and cosine functions listed in Eq. (A9) of Ref. 14. The indices l_1 and l_2 refer to θ_2 and θ_1 dependence, whereas l runs from 0 to the sum of l_1 and l_2. The expansion coefficients $v_{l_1 l_2 l}$ were obtained through a least-squares fit on the random grid of 3000 angular geometries at each intermolecular separation (i.e., each $v_{l_1 l_2 l}(R)$ is in a tabular form at this point). All terms up to $l_1 = 10$, $l_2 = 6$, and $l = 16$ were included, resulting in 142 terms in Eq. (13), as in Ref. 15. The RMSE value was found to be lower than 0.1 cm^{-1} for all intramonomer coordinates (r, s) when $R \gtrsim 6$ bohr. For (r, s) points close to (r_c, s_c), the RMSE was lower than 0.1 cm^{-1} already for $R \gtrsim 4.5$ bohr. We then selected only the most significant terms using a self-consistent Monte Carlo error estimator (defined in Ref. 16), resulting in a final set of 60 expansion functions with indices up to $l_1 = 7$, $l_2 = 4$, and $l = 11$. The Monte Carlo approach also allows us to estimate the mean error of the expansion coefficients $v_{l_1 l_2 l}(R)$ as smaller than 0.1 cm^{-1} for $R \gtrsim 5$ bohr. Finally, a cubic spline radial interpolation of the coefficients $v_{l_1 l_2 l}(R)$ was performed over the whole R range and it was smoothly connected using a switching function to standard extrapolations (exponential and power laws at the short and long-range, respectively) in order to provide continuous radial expansion coefficients suitable for the scattering calculations. Details of the switch function can be found

in Ref. 17.

IV. VIBRATIONAL AVERAGING

In the main text, we presented scattering cross sections obtained from the vibrationally averaged potential energy surface (PES) $\langle V_{15}\rangle_0$. This averaging was over the *para* ground state rovibrational wave function, i.e., $(v_{\mathrm{H}_2} = 0, j_{\mathrm{H}_2} = 0)$. It can be noticed on Figs. 1-2 of the main text that the agreement between the full-6D treatment and the 4D calculations based on the $\langle V_{15}\rangle_0$ PES is better for para-$\mathrm{H}_2(j_{\mathrm{H}_2} = 0)$ than for ortho-$\mathrm{H}_2(j_{\mathrm{H}_2} = 1)$. As shown in the figure below, when the 6D PES is averaged over the $(v_{\mathrm{H}_2} = 0, j_{\mathrm{H}_2} = 1)$ rovibrational wave function instead of $(v_{\mathrm{H}_2} = 0, j_{\mathrm{H}_2} = 0)$, the agreement with the full-6D treatment improves significantly and the shift becomes smaller than 0.01 cm^{-1} also for ortho-H_2.

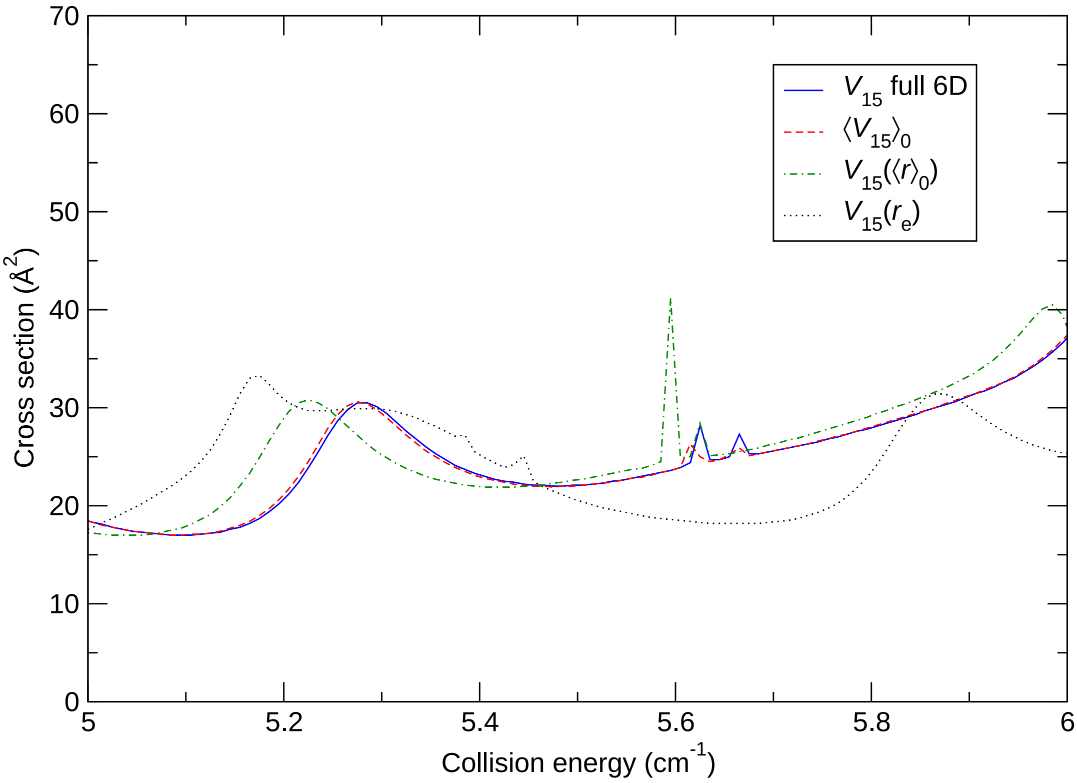

FIG. 1. Cross sections for the CO excitation $(j_{\mathrm{CO}} = 0 \to 1)$ due to ortho-$\mathrm{H}_2(j_{\mathrm{H}_2} = 1)$ as functions of the collision energy in the range 5–6 cm^{-1}. Here the full-6D PES V_{15} was averaged over the $(v_{\mathrm{H}_2} = 0, j_{\mathrm{H}_2} = 1)$ rovibrational wave function.

V. CONVOLUTION OF CROSS SECTIONS

In order to compare our scattering calculations with the measurements of Chefdeville *et al.*[18], the computed cross sections $\sigma(E)$ were convolved with the experimental collision energy spread according to:

$$\sigma_{\mathrm{conv}}(E) = \int_0^\infty \sigma(E')G_\delta(E-E')dE' \tag{14}$$

with

$$G_\delta(E) = \frac{1}{\sqrt{2\pi}\delta}\exp\left(-\frac{E^2}{2\delta^2}\right), \tag{15}$$

where $\delta(E)$ is determined by the crossed-beam experiment of Ref. 18 (Astrid Bergeat, private communication):

$$\delta(E) = \frac{1}{\sqrt{2}}(0.827 + 0.0826E - 5.24\times10^{-4}E^2 + 3.73\times10^{-6}E^3 - 9.87\times10^{-9}E^4), \tag{16}$$

for para-H_2 and

$$\delta(E) = \frac{1}{\sqrt{2}}(0.909 + 0.0833E - 5.26\times10^{-4}E^2 + 3.40\times10^{-6}E^3 - 8.15\times10^{-9}E^4), \tag{17}$$

for normal-H_2.

VI. CROSS SECTION FOR $j_{\mathrm{CO}} = 0, 1 \to 2$ EXCITATION

The cross sections for the excitation of CO ($j_{\mathrm{CO}} = 0 \to 1$) due to para- and normal-H_2 were presented in the main text. The results obtained for CO ($j_{\mathrm{CO}} = 0, 1 \to 2$) excitation due to para-$H_2(j_{H_2} = 0)$ are displayed in Fig. 2 here. The experimental data are taken from Fig. 1 of Ref. 18. The theoretical cross sections were calculated with the 6D V_{15} surface and were convolved with the experimental energy spread. The theoretical data correspond to the sum of two contributions: the excitations from the initial levels $j_{\mathrm{CO}} = 0$ and $j_{\mathrm{CO}} = 1$ with weights of 90% and 10%, respectively, based on average population of the $j_{\mathrm{CO}} = 1$ level in the experiment. We note in particular that the $j_{\mathrm{CO}} = 1 \to 2$ contribution explains the behaviour of the experimental cross section below the $j_{\mathrm{CO}} = 0 \to 2$ threshold, as pointed out in Chefdeville *et al.*[18] The agreement between theory and experiment is again very good, actually better than on Figs. 3 and 4 of the main text as most of the computed values are within the experimental error bars. The agreement is particularly impressive close to

:

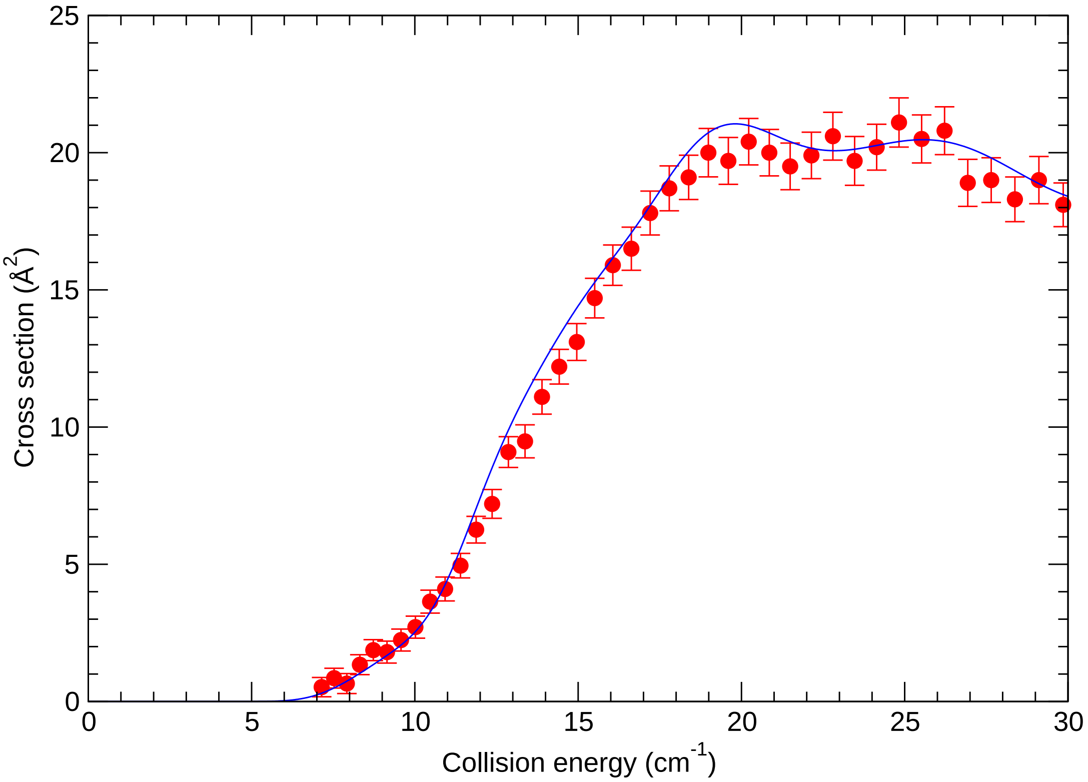

FIG. 2. Cross sections for CO excitation ($j_{CO} = 0, 1 \to 2$) due to para-H_2($j_{H_2} = 0$) as function of collision energy. Theory (blue line) is from 6D calculations with V_{15}. The experimental data are taken from Fig. 1 of Ref. 18.

the threshold, where the error bars are smallest. This improved agreement may indicate that the experimental disentanglement of different types of scattering introduces additional uncertainties not fully accounted by the error bars.

---

[1] Jankowski, P. *et al.* A comprehensive experimental and theoretical study of H_2-CO spectra. *J. Chem. Phys.* **138**, 084307 (2013).

[2] Jankowski, P. & Szalewicz, K. A new *ab initio* interaction energy surface and high-resolution spectra of the H_2–CO van der Waals complex. *J. Chem. Phys.* **123**, 104301 (2005).

[3] Jankowski, P., McKellar, A. R. W. & Szalewicz, K. Theory untangles the high-resolution infrared spectrum of the *ortho*H_2-CO van der Waals complex. *Science* **336**, 1147–1150 (2012).

[4] Kendall, R. A., Dunning, Jr., T. H. & Harrison, R. J. Electron-affinities of the 1st-row atoms revisited - systematic basis-sets and wave-functions. *J. Chem. Phys.* **96**, 6796–6806 (1992).

[5] Halkier, A., Klopper, W., Helgaker, T., Jørgensen, P. & Taylor, P. R. Basis set convergence of the interaction energy of hydrogen-bonded complexes. *J. Chem. Phys.* **111**, 9157–9167 (1999).

[6] Leforestier, C., Szalewicz, K. & van der Avoird, A. Spectra of water dimer from a new *ab initio* potential with flexible monomers. *J. Phys. Chem.* **137**, 014305 (2012).

[7] Garberoglio, G., Jankowski, P., Szalewicz, K. & Harvey, A. H. Second virial coefficients of H_2 and its isotopologues from six-dimensional potential. *J. Chem. Phys.* **137**, 154308 (2012).

[8] Jankowski, P. *et al.* Ab initio water pair potential with flexible monomers. *J. Phys. Chem. A* **119**, 2940–2964 (2015).

[9] Szalewicz, K., Murdachaew, G., Bukowski, R., Akin-Ojo, O. & Leforestier, C. Spectra of water dimer from *ab initio* calculations. In Maroulis, G. & Simos, T. (eds.) *Lecture Series on Computer and Computational Science: International Conference of Computational Methods in Science and Engineering (ICCMSE 2006)*, vol. 6, 482–491 (Brill Academic Publishers, Leiden, 2006).

[10] Bukowski, R. *et al.* Intermolecular potential of carbon dioxide dimer from symmetry-adapted perturbation theory. *J. Chem. Phys.* **110**, 3785–3803 (1999).

[11] Jankowski, P. & Szalewicz, K. *Ab initio* potential energy surface and infrared spectra of H_2–CO and D_2–CO van der Waals complexes. *J. Chem. Phys.* **108**, 3554–3565 (1998).

[12] Tang, K. T. & Toennies, J. P. An improved simple model for the van der waals potential based on universal damping functions for the dispersion coefficients. *J. Chem. Phys.* **80**, 3726–3741 (1984).

[13] Jeziorski, B., Moszynski, R. & Szalewicz, K. Perturbation theory approach to intermolecular potential energy surfaces of van der waals complexes. *Chem. Rev.* **94**, 1887–1930 (1994).

[14] Green, S. Rotational excitation in H_2-H_2 collisions - Close-coupling calculations. *J. Chem. Phys.* **62**, 2271–2277 (1975).

[15] Wernli, M. *et al.* Improved low-temperature rate constants for rotational excitation of CO by H_2. *Astron. Astrophys.* **446**, 367–372 (2006).

[16] Rist, C. & Faure, A. A Monte Carlo error estimator for the expansion of rigid-rotor potential energy surfaces. *J. Math. Chem.* **50**, 588–601 (2012).

[17] Valiron, P. *et al.* R12-calibrated H_2O-H_2 interaction: Full dimensional and vibrationally averaged potential energy surfaces. *J. Chem. Phys.* **129**, 134306 (2008).

[18] Chefdeville, S. *et al.* Experimental and theoretical analysis of low-energy CO + H_2 inelastic collisions. *Astrophys. J. Lett.* **799**, L9 (2015).

TABLE I. The values of $E_{\text{int,B}}$, $E_{\text{int,hyb}}$, and $E_{\text{int,H}}$ are presented in the rows denoted by A, B, and C, respectively, for two intermolecular sets of parameters $\boldsymbol{X} = (7.0, 0°, 0°, 0°)$ and $\boldsymbol{X} = (8.0, 0°, 180°, 0)°$, and a grid of the intramolecular coordinates $\boldsymbol{Y} = (r, s)$. The percentage error of $E_{\text{int,B}}$ and $E_{\text{int,hyb}}$, with respect to $E_{\text{int,H}}$, are given in the parenthesis. The reference values of $E_{\text{int,B}}$ and $E_{\text{int,hyb}} = E_{\text{int,H}}$ calculated for $(r_c, s_c) = (1.474, 2.165)$ bohr are -74.809 cm^{-1} and -75.314 cm^{-1} for $(7.0, 0°, 0°, 0°)$, and -91.318 cm^{-1} and -94.045 cm^{-1} for $(8.0, 0°, 180°, 0°)$, respectively. All energies are given in cm^{-1} and distances in bohr.

$r\backslash s$	1.90	1.99	2.13	2.30	2.45	
			$(7.0, 0°, 0°, 0°)$			
0.95	-42.402(-1.37%)	-45.884(-1.11%)	-51.339(-0.67%)	-57.701(-0.10%)	-62.770(0.41%)	A
	-42.907(-0.20%)	-46.389(-0.02%)	-51.844(0.31%)	-58.206(0.78%)	-63.275(1.21%)	B
	-42.992	-46.400	-51.686	-57.757	-62.516	C
1.20	-49.974(-1.66%)	-54.760(-1.33%)	-62.294(-0.78%)	-71.130(-0.07%)	-78.232(0.54%)	A
	-50.479(-0.67%)	-55.265(-0.42%)	-62.799(0.03%)	-71.635(0.63%)	-78.737(1.19%)	B
	-50.818	-55.497	-62.783	-71.183	-77.811	C
1.40	-54.743(-1.89%)	-60.636(-1.48%)	-69.949(-0.84%)	-80.928(-0.02%)	-89.819(0.70%)	A
	-55.248(-0.98%)	-61.141(-0.66%)	-70.454(-0.12%)	-81.433(0.61%)	-90.324(1.26%)	B
	-55.796	-61.550	-70.539	-80.943	-89.197	C
1.67	-57.756(-2.26%)	-65.125(-1.73%)	-76.848(-0.91%)	-90.787(0.11%)	-102.209(0.99%)	A
	-58.261(-1.40%)	-65.630(-0.97%)	-77.353(-0.26%)	-91.292(0.66%)	-102.714(1.49%)	B
	-59.089	-66.272	-77.552	-90.691	-101.207	C
2.05	-50.309(-3.72%)	-59.418(-2.75%)	-74.092(-1.39%)	-91.831(0.16%)	-106.689(1.45%)	A
	-50.814(-2.76%)	-59.923(-1.93%)	-74.597(-0.72%)	-92.336(0.71%)	-107.194(1.93%)	B
	-52.254	-61.100	-75.136	-91.688	-105.167	C
			$(8.0, 0°, 180°, 0°)$			
0.95	-59.886(-1.95%)	-57.464(-2.13%)	-53.415(-2.45%)	-48.118(-2.89%)	-43.168(-3.35%)	A
	-62.613(2.51%)	-60.191(2.51%)	-56.142(2.53%)	-50.845(2.61%)	-45.895(2.76%)	B
	-61.077	-58.717	-54.757	-49.550	-44.662	C
1.20	-82.361(-2.03%)	-78.528(-2.24%)	-72.136(-2.61%)	-63.797(-3.16%)	-56.044(-3.75%)	A
	-85.088(1.21%)	-81.255(1.15%)	-74.863(1.07%)	-66.524(0.98%)	-58.771(0.94%)	B
	-84.069	-80.330	-74.071	-65.876	-58.225	C
1.40	-101.678(-2.12%)	-96.518(-2.34%)	-87.923(-2.74%)	-76.733(-3.35%)	-66.360(-4.03%)	A
	-104.405(0.51%)	-99.245(0.42%)	-90.650(0.28%)	-79.460(0.09%)	-69.087(-0.09%)	B
	-103.877	-98.830	-90.401	-79.392	-69.150	C
1.67	-128.156(-2.24%)	-120.929(-2.48%)	-108.914(-2.94%)	-93.310(-3.66%)	-78.896(-4.51%)	A
	-130.883(-0.16%)	-123.656(-0.29%)	-111.641(-0.51%)	-96.037(-0.84%)	-81.623(-1.21%)	B
	-131.087	-124.010	-112.215	-96.855	-82.619	C
2.05	-161.313(-2.74%)	-150.664(-3.07%)	-133.001(-3.69%)	-110.138(-4.73%)	-89.111(-6.05%)	A
	-164.040(-1.10%)	-153.391(-1.32%)	-135.728(-1.72%)	-112.865(-2.37%)	-91.838(-3.17%)	B
	-165.866	-155.437	-138.103	-115.604	-94.845	C

TABLE II. List of the polynomials w_i in variables r and s corresponding to the intramonomer distances in the H_2 and CO molecules, respectively. The values of the reference separations are $r_c = 1.474$ bohr and $s_c = 2.165$ bohr.

i	$w_i(r,s)$
1	1
2	$(r - r_c)$
3	$(s - s_c)$
4	$(r - r_c)^2$
5	$(r - r_c)(s - s_c)$
6	$(s - s_c)^2$
7	$(r - r_c)^3$
8	$(r - r_c)^2(s - s_c)$
9	$(r - r_c)(s - s_c)^2$
10	$(s - s_c)^3$
